# Acceptance of Digital Health Technologies in Palliative Care Patients

**DOI:** 10.1089/pmr.2023.0062

**Published:** 2024-01-12

**Authors:** Stefan Wicki, Ian C. Clark, Manuel Amann, Sebastian M. Christ, Markus Schettle, Caroline Hertler, Gudrun Theile, David Blum

**Affiliations:** ^1^ Department of Radiation Oncology, Competence Center Palliative Care, University Hospital and University of Zurich, Zurich, Switzerland.; ^2^ Department of Radiation Oncology, University Hospital and University of Zurich, Zurich, Switzerland.

**Keywords:** AI, digital health technologies, ePROM, palliative care, VR, wearables

## Abstract

**Background:**

Digital health technologies have potential to transform palliative care (PC) services. The global aging population poses unique challenges for PC, which digital health technologies may help overcome. Evaluation of attitudes and perceptions combined with quantification of prior use habits favor an understanding of psychological barriers to PC patient acceptance of digital health technologies including artificial intelligence (AI).

**Objectives:**

We aimed to evaluate the attitudes and perceptions of PC patients regarding a broad range of digital health technologies used in their routine monitoring and treatment and identify barriers to use.

**Methods:**

We used a 39-item questionnaire to evaluate acceptance and use of smartphone-based electronic patient report outcome measures, wearables, AI, data privacy, and virtual reality (VR) in 29 female and male PC inpatients.

**Results:**

A majority of patients indicated an interest in (69.0%) and positive attitude toward (75.9%) digital health technologies. Nearly all (93.1%) patients believe that digital health technologies will become more important in medicine in the future. Most patients would consider using their smartphone (79.3%) or wearable (69.0%) more often for their health. The most feasible technologies were smartphones, wearables, and VR. Barriers to acceptance included unfamiliarity, data security, errors in data interpretation, and loss of personal interaction through AI.

**Conclusion:**

In this patient survey, acceptance of new technologies in a PC patient population was high, encouraging its use also at the end-of-life.

## Introduction

### Background

Digital health technologies include telehealth, applications, wearable sensors, artificial intelligence (AI), and human–machine interaction and together have potential to transform health care services in palliative care (PC).^1^ The global aging population will be predictably accompanied by disability and disease and poses unique challenges for PC services which could be met with digital health technologies.^2^ Digital health technologies are increasingly utilized in PC.^3^ The World Health Organisation (WHO) released digital health implementation guidelines and suggested that digital health be a fundamental health priority to improve global health.^4–6^ A recent Delphi study^7^ identified research priorities in digital health technologies in PC, which included investigating barriers to accessibility and acceptance of digital health technologies among PC patients. Evaluation of attitudes and perceptions combined with quantification of prior use habits of digital health technologies in PC patients including AI favor an understanding of these psychological barriers and could help to overcome them.

The COVID-19 pandemic underscores the unpredictable nature of health care management and the acute and urgent need for remote digital solutions.^8^ Digital health technologies designed to mitigate the effects of isolation were shown to be effective, and utilizing digital technologies during the pandemic emerged as a recommendation in one review.^9^ Multiple studies^10–12^ have shown that electronic patient reported outcome measures (ePROMs) can improve communication between patients and clinicians and alert clinicians to the acute needs of patients during at-home care. Whereas ePROMs provide subjective health data, wearables, such as smartwatches, capture objective data.^13^

### Objectives

We developed a novel questionnaire evaluating the attitudes and perceptions of a variety of digital health technologies used in routine monitoring and treatment of PC patients and asked patients to estimate their habitual use of a selection of common consumer digital technologies (i.e., smartphone, personal computer, tablet, and smartwatch) as applied to their health and medical care. We additionally asked these patients to evaluate the perceived strengths and weaknesses, as well as potential benefits and risks, of such technologies. Due to the recent emergence of revolutionary AI, such as the highly advanced Natural Language Processing model Chat Generative Pre-Trained Transformer (Chat GPT)^14^ and the ethical and practical considerations associated with implementation of AI in health care,^15^ we specifically inquired about patient–AI interactions in the context of their own medical care. We defined acceptance in the context of technology utilization: approval, favorable reception, and continued use of newly introduced device or systems.^16,17^

## Methods

### Developing a questionnaire

Via research group discussion and database research, we developed a pen-and-paper questionnaire measuring the strength of acceptance of digital health technologies in PC patients. We decided to create our own questionnaire to specifically tailor it to our ideas and resources. To our knowledge, no questionnaire was available that fit our needs.

### Pilot study

We piloted it in two male and three female PC inpatients (59.8 ± 14 years) at the PC ward of the University Hospital Zurich (UHZ) in October 2020. We revised it based on their feedback and PC expert input. The final questionnaire included 39 questions, 17 Likert-type with 5 response levels, 9 open-ended regarding risk–benefits or reasoning behind a response, 5 multiple choice (with a write-in response option), and 3 dichotomous. If a patient responded that they possessed a particular device (e.g., smartphone), we asked them to quantitatively estimate (e.g., hours/week) habitual use of the device for health purposes. We divided the questionnaire by topic, including apps, wearables, AI, data privacy, and virtual reality (VR). A brief explanation introduced each topic.

### Setting and inclusion criteria

We implemented the questionnaire at the PC wards of the UHZ and the Clinic Susenberg Zurich (CSZ) between January 1, 2021 and April 30, 2021. Inclusion criteria: age ≥18 years; PC inpatient status regardless of diagnosis (oncological and nononcological) at either clinic; German comprehension; cognitive adequacy. Exclusion criteria: inability or unwillingness to provide informed consent.

### Recruitment

Our sample comprised 29 adult patients (69.5 ± 11.8 years) of both sexes (16 female, 13 male), 26 with oncological diagnosis. Patients enrolled between January 1, 2021 and April 30, 2021 and were informed of the study purpose, procedures, duration (ca. 45 minutes), and that they may withdraw at any time without justification or repercussion. Between these dates, ca. 80 and 60 potentially eligible patients were admitted to the UHZ and CSZ PC wards, respectively. Of the total 140 patients, ca. 40% were unconscious or dying, 10% indicated language difficulty and another 10% were not interested. We screened the remaining ca. 60 patients at these respective sites, of which 18 and 11 elected to participate. Our sample was purposive, such that only those remaining patients who indicated sufficient health and a willingness to participate were included. This study met criteria for exemption as reviewed by the Cantonal Ethics Commission of the Canton of Zurich (BASEC-Nr. 2021-00093). All patients provided prior written informed consent.

### Procedures

Patients completed the questionnaire in the hospital independently or with a researcher. In either case, patients could address any questions to a researcher. We collected demographic data from their medical record, including age, gender, and primary diagnosis.

### Statistical analysis

All analyses and data processing steps were performed in the statistical programming language *R* (version 4.2.0; R Foundation for Statistical Computing).^18^ Likert scale data were coded and analyzed as ordinal variables; dichotomous (i.e., “Yes,” “No”) data were analyzed as nominal variables. Quantitative data (i.e., age and time spent using a device) were analyzed as continuous variables. Benjamini–Hochberg correction was used for *post hoc* multiple comparisons. An alpha of 0.05 was set for all statistical tests.

## Results

### Experience and interest in modern technologies

**FIG. 1.**
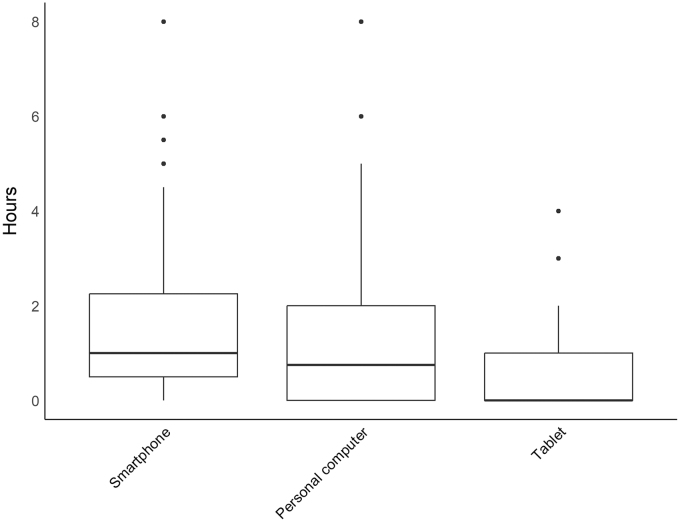
Hours per day by device type (*n* = 28 due to a missing value). Box plots show median, first and third quartiles and whiskers (±1.5 × interquartile range). Individual points indicate outlying values.

Patient demographics are displayed in Table 1. Twenty patients (69.0%) were “interested” or “rather interested” in digital health technologies such as smartphones, computers, smartwatches, or VR-glasses, and 22 patients (75.9%) had a “positive” or “rather positive” general attitude toward them. Whereas 12 patients (41.4%) reported they had “a lot” of experience with smartphones and computers, 10 patients (34.5%) had “little to no experience.” Twenty-five patients (86.2%) owned a smartphone, 24 patients (82.8%) owned a computer, and 14 patients (48.3%) owned a tablet. Only one patient (3.4%) owned a smartwatch and no patients owned VR-glasses. One patient did not respond (3.4%). Figure 1 shows the average amount of time in hours per day using a device (omitting smartwatch) for any purpose. Kruskal–Wallis test showed that patients (*n* = 28 due to missing value) did not spend a statistically equal amount of time on all three devices (chi-square = 11.89, df = 2, *p* < 0.01). The most time was spent on the smartphone (1.95 ± 2.16 hours), followed by personal computer (1.49 ± 1.99 hours), and the least time was spent on the tablet (0.61 ± 1.03 hours). *Post hoc* testing (Mann–Whitney *U* test) revealed only time spent on the personal computer and tablet to differ (0.88 hours) statistically, however (*U* = 517.5, *p* < 0.05).

**Table 1.** Demographics

	Total	Women	Men
Age (year)	69.5 ± 11.8	71.4 ± 11.27	67.1 ± 12.45

Variable			
*n* (%)	29 (100)	16 (55.2)	13 (44.8)
Clinic			
UHZ	18 (62.1)	7 (24.1)	11 (37.9)
CSZ	11 (37.9)	9 (31.0)	2 (6.9)
Questionnaire			
Independent	15 (51.7)	7 (24.1)	8 (27.6)
Interview	14 (48.3)	9 (31.0)	5 (17.2)

Age data are presented as mean ± standard deviation.

CSZ, Clinic Susenberg Zurich; UHZ, University Hospital Zurich.

Twenty-seven patients (93.1%) thought digital health technologies will gain importance in medicine, and 19 (65.5%) valued this “positively” or “rather positively.” Only one patient (3.4%) valued this “rather negatively.” One patient did not respond (3.4%).

### Applications

Eight patients (27.6%) reported already using their smartphone for medical purposes. Of these, three used an application (app) for medication reminders regarding timing and dose, two to access general information about their illness and one for therapeutic advice based on self-reported symptoms. One used an app for pacemaker information, and finally one connected to a general practitioner and home care providers.

Twenty-three patients (79.3%) reported they would consider increasing their smartphone-usage for medical purposes. Thirteen patients would consider using an app to remind them when and how to take their medication. Seventeen patients would consider using an app to access general information about their disease. Smaller numbers of patients would consider using an app to connect with other patients with similar illnesses (7), use an app to record self-reported symptoms (4), or receive therapy advice based on self-reported symptoms (8).

**FIG. 2.**
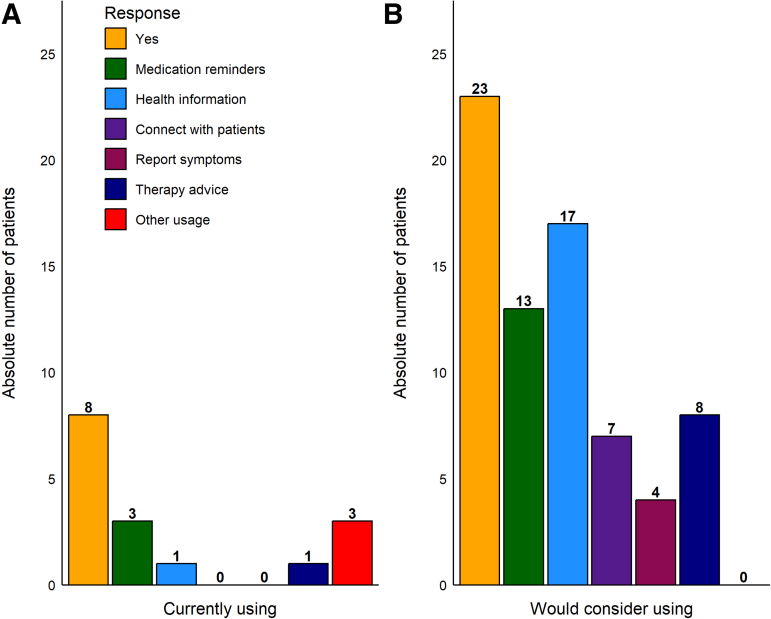
Patient use of smartphones for medical purposes: present and future. Smartphone medical use categories are color-coded. Yellow column (“Yes”) indicates that a patient is using **(A)** or would use **(B)** their smartphone for medical purposes. Numerals above bars indicate number of responses per category. Data from 8 patients (11 missing values) contributed to the “Currently using” plot **(A)**, whereas data from 23 patients (6 missing values) contributed to the “Would consider using” plot **(B)**. Responses to a given smartphone medical use category were not mutually exclusive, such that data from a single patient could contribute to several categories, indicating that the patient is already now or would in the future consider using the smartphone for multiple different health categories. The response category “Other usage” was open format and patients indicated digital health usage in their own words.

Reasons mentioned for rejection of apps included a personal lack of smartphone skills, doubts about app quality, fear of mistakes, and misconceptions in interpreting the data, and fear of reduction of personal interaction with clinicians. Figure 2 shows which medical purposes patients are currently using their smartphones for and which purposes they would consider in the future. Fisher's exact test showed patients would be significantly (*p* < 0.05) more willing to use their smartphone for medical purposes in the future than at present.

Patients identified increased treatment efficiency and early detection of health deterioration as the potential benefits of app use for medical purposes. The risks were insufficient data security, fear of reduction of personal interaction with clinicians, and fear of mistakes and misconceptions in interpreting the data.

### Wearables

Twenty patients (69.0%) reported that they would consider using a wearable for monitoring their health, although they cited their immobility and questionable benefit from monitoring as reasons not to. Three patients did not respond (10.3%). The benefits for using wearables for monitoring were increased accuracy through more data, early detection of health deterioration, and encouraging personal responsibility for health. The perceived risks were again insufficient data security and fear of mistakes and misconceptions in data interpretation.

### Artificial intelligence

Most patients (93.1%) reported that personal interaction with their clinician during treatment is “important” or “rather important.” More than half (55.2%) reported concern that there would be a reduction of personal interaction with clinicians due to increasing implementation of AI in health care.

One section of the questionnaire required patients to imagine themselves in each of the following three hypothetical situations in which AI replaced clinicians:

- Situation 1: You have a headache at home and consider seeing a doctor about it. Would you consider using an app to get medical advice in this situation first?
- Situation 2: You have a routine consultation at the hospital. Would you consider filling out an online questionnaire about your health, which would then be provided to your attending clinician, before seeing said clinician?
- Situation 3: Would you consider being diagnosed/treated exclusively by a computer algorithm/AI regarding a medical problem?

**FIG. 3.**
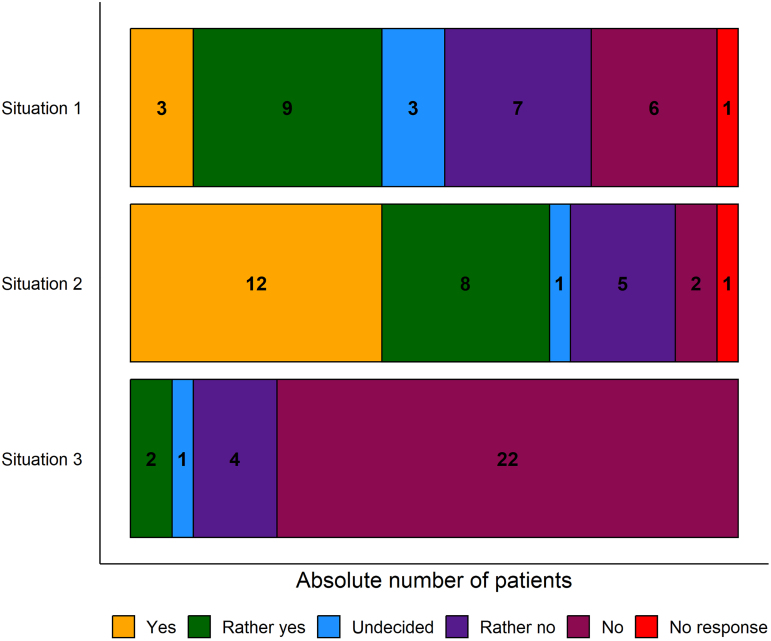
Responses to three hypothetical situations AI. Five response-level Likert scale data are color-coded and overlaid by numerals indicating absolute number of patients per response. Data for 29 participants are displayed; two missing values are presented in red (“No response”). AI, artificial intelligence.

As Figure 3 shows, the distribution of responses was not equal across the three hypothetical scenarios (Fisher's exact test, *p* < 0.001). Responses to consulting an app for advice (Situation 1) was most heterogeneous, with nearly as many patients endorsing “Yes” (3) or “Rather yes” (9) as “No” (7) or “Rather no” (6); this situation contained the most undecided responses (3) of the three. Filling out an online questionnaire (Situation 2) was met with broad acceptance, with patients overwhelmingly endorsing “Yes” (12) or “Rather yes” (8). Diagnosis or treatment via AI (situation 3) was roundly rejected, such that the majority responded with “Rather no” (4) or “No” (22) and no patients responded with “Yes.”

If a patient responded negatively (i.e., “Rather no” or “No”) to a hypothetical situation, the patient was prompted to give their rationale. For situation 1, rationale included not needing assistance in the case of a headache, preferring personal evaluation and treatment through their clinician and not trusting an algorithm because of the complexity of a health problem. Responses to situation 2 included the need for personal interaction, the general oddity of the situation and fear of technical difficulties. For situation 3, patient rationale included ethical reasons, lack of empathy by the AI, importance of personal interaction, not trusting AI because of a complex health problem as well as fear of mistakes and misconceptions in interpreting the data.

With respect to risks and benefits, patients found that AI-supported decision-making in medicine would benefit better diagnosis and therapy, improve medical practice through collection of objective data, and reduce clinician workload. Risks of AI-supported decision-making included a lack of empathy, a lack of human supervision, a loss of humanity, fear of mistakes, and misconceptions in interpreting the data.

### Data privacy

**FIG. 4.**
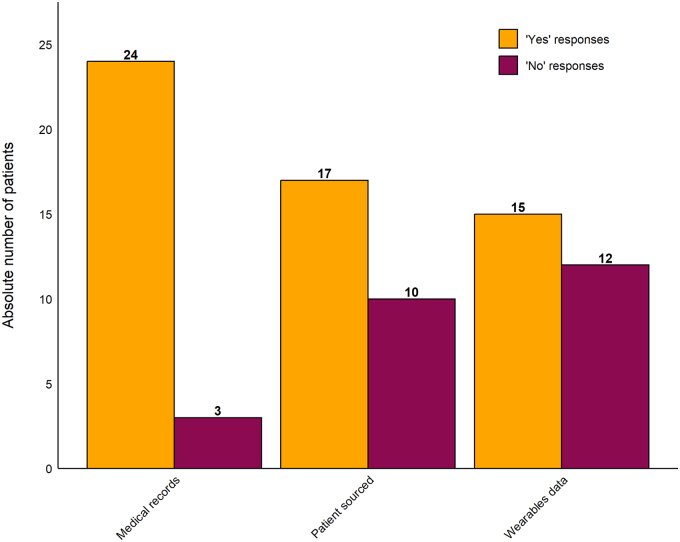
Willingness to share personal health data with a hypothetical algorithm to aid AI-supported decision-making by data source. Twenty-seven patients (two missing values) contributed data to this figure, such that two patients declined to provide a response, either for or against sharing data, in any source category. “Medical records”: data from a patient's medical records; “Patient sourced”: data the patient reports about their well-being or symptoms (for example via app); “Wearables”: data from a wearable device. AI, artificial intelligence.

A majority (69.0%) reported their health data security is “important” or “rather important.” Figure 4 identifies which sources of health data patients would be willing to share with a hypothetical algorithm to aid in AI-supported decision-making. Of those who responded to questions pertaining to data source, most would be willing to provide medical records data (24), followed by patient-reported well-being or symptoms data (17). About half of patients would share their wearable-derived health data (15). Responses were not equally distributed (chi-square = 7.75, df = 2, *p* < 0.05); however, of the three categories, only in the “Medical records” category, there were significantly more patients willing to share (24) than not share (3) their data (*post hoc* test of standardized residuals, *p* < 0.05). Patients reported that big data would benefit better diagnosis and therapy, improve medical practice through collection of objective data and support future research. The primary risk noted was data security.

### Virtual reality

No patient possessed a VR system/apparatus and only 4 (13.8%) had used VR before, yet half (48.3%) reported they would use VR in a medical setting. Patients were asked about virtual travel destinations and five wished to visit their “favorite place,” seven a “real place you'd always wanted to visit,” and one an “imaginary place.” These were multiple-choice options; no patient elected to suggest a destination of their own. Typical reasons for a lack of interest in VR included fear of nausea and dizziness and not recognizing the point of it. Of those who responded to the question of risks or benefits (8), most (5) saw VR as a benefit and cited possible mood improvement, less monotony during hospitalizations and distraction from illness. The primary risk was loss of a sense of reality.

## Discussion

This study investigated the acceptance of digital health technologies in 29 PC patients and could demonstrate strong acceptance of digital health technologies through several different outcomes examining acceptance from different perspectives. A majority of patients indicated an interest in (69.0%) and positive attitude toward (75.9%) digital health technologies in general. Nearly all (93.1%) patients believed that digital health technologies will become more important in medicine and only one patient considered this an unfavorable development. This optimism for a hypothetical future improvement of health care through digital technologies among older adults has been observed elsewhere^19^ and supports the notion of acceptance.

General prior experience with digital health technologies was variable, such that the proportion of those with a lot (41.4%) versus a little (34.5%) was about even. Perhaps predictably so, smartphones and personal computers were the most popular devices to own, such that about 90% of patients owned either. These observations are broadly in line with trends^20^ among the general older adult population for smartphone^21^ and personal computer^22^ use. The average number of hours spent on either the smartphone (1.95 ± 2.16 hours) or personal computer (1.49 ± 1.99 hours) reflects their popularity compared to tablet and smartwatch and fits into screen time exposure patterns of older adults surveyed elsewhere.^23^ Older adults are not the primary consumers of wearables generally,^24^ and they have a different function compared to the smartphone and personal computer, which may have contributed to their unpopularity in our sample in terms of ownership, but this apparently did not diminish a willingness to consider future use. Whereas only few patients already use digital technologies such as their smartphone for their health, our study shows that the willingness to increase usage for health purposes is present. Most patients sampled would be willing to use their smartphone more for health purposes, particularly for reminders about medicine-intake or the documentation of self-reported symptoms. While almost none of the patients had experience with wearables and VR, many reported a willingness to try them for medical purposes. Acceptance of wearables^25^ and VR^26,27^ in the PC population was shown elsewhere. The most feasible technologies in terms of the proportion of patients indicating that they would consider using a given technology as part of their medical care were smartphones (79.3%), wearables (69.0%), and VR (48.3%).

Nearly all (93.1%) patients found personal interaction with clinicians during their treatment to be important. This is reflected in the literature such that increased access to and contact with clinicians via an app was identified as its most valuable feature by outpatient cancer patients.^28^ Similarly, the majority of a sample of hospice patients indicated that the single best feature of telehospice care was immediate access to their clinician.^29^ Patients felt that telehealth improved their access to clinicians.^30^ Usefulness in communicating with a clinician was cited as a benefit of a web-based portal among older adults with multiple chronic conditions.^31^

Barriers to use of digital health technology in this study have been observed in the literature on older adults,^30,31^ such as unfamiliarity with the technology^32^ and concerns pertaining to data security and mistakes and misconceptions in interpreting data.^33^ While most (82.8%) patients in our sample were willing to share their medical records data in hope of an improved computer-aided decision-making, there was an unwillingness to share other data sources such as wearable data. Questionable advantages of the technology was also reported elsewhere.^30,32,33^

More than half (55.2%) of the patients in our sample were concerned that AI might replace personal interaction with their clinician. Distrust of AI, lack of empathy, and mistakes and misconceptions in interpreting data were present in our sample. Patients seem to view AI best when it is supplementary to human decision-making as it pertains to their diagnosis and treatment, a sentiment that is echoed in a report of patients' perception of AI-performance in evaluating radiology imaging data.^34^ Our sample's concern that accuracy may be compromised is also found in this literature.^34^ Communicating to patients that there is human oversite might help overcome barriers to acceptance of AI.

A limitation of our study is that our questionnaire has not been validated. Our small purposive sample (*n* = 29) should be viewed critically when extrapolating findings to the PC patient population generally. We did not incorporate the primary diagnosis into any analysis, qualitative or otherwise. It is not possible to rule out response bias.

## Conclusions

Our study demonstrates strong acceptance of digital health technologies in our 29 surveyed PC patients. We observed that many participants are interested in and optimistic about the prospects of digital health technologies and are willing to increase their usage of such for health purposes, which include smartphones, wearables, and VR, despite some unfamiliarity. Frequently mentioned concerns among our patients were a decrease of human interaction in health care, data security, and a lack of trust in AI. Therefore, it is essential that these concerns are addressed when considering the involvement of digital technologies in a patient's medical care, particularly AI. Digital health technologies should be used as a supplement to established medical care rather than as a replacement. When implemented and used correctly, digital health technologies have the potential to improve the medical care of PC patients and rise to the challenges posed by a rapidly aging global population. Further research is warranted to extend and confirm these findings in larger samples also ideally integrating individual differences, medical diagnosis, and the practical, psychosocial, and spiritual demands specific to the PC-setting.

## Abbreviations Used

AI: artificial intelligence
CSZ: Clinic Susenberg Zurich
ePROM: electronic patient reported outcome measures
PC: palliative care
UHZ: University Hospital Zurich
VR: virtual reality
WHO: World Health Organisation
